# Network Structure Engineering of Organosilica Membranes for Enhanced CO_2_ Capture Performance

**DOI:** 10.3390/membranes12050470

**Published:** 2022-04-27

**Authors:** Qiwei Jiang, Meng Guo

**Affiliations:** 1 Wuxi Ginkgo Plastic Industry Co., Ltd., Heqiao Town, Yixing, Wuxi 214216, China; jqwei1983@163.com; 2 Jiangsu Key Laboratory of Advanced Catalytic Materials and Technology, School of Petrochemical Engineering, Changzhou University, Changzhou 213164, China

**Keywords:** organosilica membranes, CO_2_ capture, calcination temperatures, pore size tailoring

## Abstract

The membrane separation process for targeted CO_2_ capture application has attracted much attention due to the significant advantages of saving energy and reducing consumption. High-performance separation membranes are a key factor in the membrane separation system. In the present study, we conducted a detailed examination of the effect of calcination temperatures on the network structures of organosilica membranes. Bis(triethoxysilyl)acetylene (BTESA) was selected as a precursor for membrane fabrication via the sol-gel strategy. Calcination temperatures affected the silanol density and the membrane pore size, which was evidenced by the characterization of FT-IR, TG, N_2_ sorption, and molecular size dependent gas permeance. BTESA membrane fabricated at 500 °C showed a loose structure attributed to the decomposed acetylene bridges and featured an ultrahigh CO_2_ permeance around 15,531 GPU, but low CO_2_/N_2_ selectivity of 3.8. BTESA membrane calcined at 100 °C exhibited satisfactory CO_2_ permeance of 3434 GPU and the CO_2_/N_2_ selectivity of 22, displaying great potential for practical CO_2_ capture application.

## 1. Introduction

The excessive emission of CO_2_ molecules has presented serious problems in the ecological environment. It is very necessary to develop novel and effective methods to control CO_2_ emission [1,2]. To keep a temperature rise within 1.5 °C, greenhouse gas emissions must be halved by 2030, and a further emission reduction of 29 billion to 32 billion tons is required [3]. The emission of CO_2_ mainly comes from the decomposition, fermentation, and pyrolysis of organic matter and the combustion of fossil energy. Specifically, CO_2_ (CO_2_/N_2_ mixtures) produced by the combustion of fossil energy accounts for the vast majority. How to capture and concentrate CO_2_ after pretreatment and further convert and utilize high-purity CO_2_ is a huge challenge for environmental protection and economic benefits [4,5].

The CO_2_ capture and removal are key issues in the process of CO_2_ emission reduction. The ethanolamine (solvent) absorption method currently used in industry has a high CO_2_ adsorption capacity. Nevertheless, its absorption capacity is related to the CO_2_ dissolution rate and solvent regeneration performance. The complex process flow, huge energy consumption, and high operating costs restrict further application. Effective and energy-efficient separation strategies must be developed urgently and utilized for CO_2_ capture [6].

Membrane separation technology has significant advantages of saving energy and reducing consumption [7,8]. It is one of the common technologies that support major issues in the fields of water resources, energy, transformation and upgrading of traditional industries, and environmental pollution control [9]. Therefore, membrane separation technology is known as the most strategic new technology for the process industry in the 21st century. Over the past few decades, many kinds of membranes, such as inorganic, organic, and organic–inorganic hybrid membranes, were extensively studied for enhancing CO_2_ capture efficiency [7,10,11,12]. Among them, organosilica membranes with high hydrothermal stability and excellent molecular sieving properties have attracted great interest and demonstrated great advantages in CO_2_ capture [8,13]. For research, improving CO_2_ selectivity and maintaining CO_2_ permeance are considered to be more innovative work. However, high CO_2_ permeance is more important than high CO_2_ selectivity in industrial application [14]. It has been reported that increasing CO_2_ selectivity above 30 does not significantly reduce operating costs, but increasing CO_2_ permeance can effectively control costs [14]. Therefore, it is very necessary to develop organosilica membranes with ultra-high permeance for the CO_2_ capture process.

Bis(triethoxysilyl)ethane (BTESE), a classic organosilica precursor, was extensively studied in the development of organosilica membranes for the application of pervaporation, reverse osmosis, and gas separation [15,16,17,18,19]. In light of CO_2_ separation, Yu et al. fabricated BTESE membranes utilizing BTESE sols synthesized in acidic pH via the pH-swing strategy [20]. The pH-swing method-derived BTESE sols have a larger size, which further restricts the penetration of BTESE sols during the membrane fabrication process. As a result, the BTESE membrane exhibited high CO_2_/N_2_ and CO_2_/CH_4_ selectivity around 28 and 90, respectively. Nevertheless, the low CO_2_ permeance of 1 × 10^−7^ mol m^−2^ s^−1^ Pa^−1^ (300 GPU, 1 GPU = 3.348 × 10^−10^ mol m^−2^ s^−1^ Pa^−1^) still needs to be further improved [21]. Considering the cost efficiency in practical application, the membranes with CO_2_ permeance higher than 1000 GPU and CO_2_/N_2_ selectivity larger than 20 can satisfy industrial requirements [22].

In contrast to ethane-bridged BTESE membranes, the acetylene-bridged bis(triethoxysilyl)acetylene (BTESA) membranes demonstrated much higher gas permeance profiting from enlarged membrane pore sizes [23]. BTESA membranes calcined at 300 °C displayed potential in C_3_H_6_/C_3_H_8_ separation with a C_3_H_6_ permeance of 1~2 × 10^−7^ mol m^−2^ s^−1^ Pa^−1^ and C_3_H_6_/C_3_H_8_ selectivity of 11~14 [23]. Even though BTESA membranes also showed ultrahigh CO_2_ permeance, their CO_2_/N_2_ selectivity was only 7–13, which should be carefully treated and enhanced to satisfy practical requirements [13]. In previous work, (3-aminopropyl)triethoxysilane (APTES) was incorporated into BTESA networks via co-polymerization reactions. The incorporation of amino groups indeed enhanced CO_2_-philic properties and well controlled the pore sizes of composite membranes [13]. These composite membranes achieved an unprecedented CO_2_ permeance ranging from 2550 to 3230 GPU and a CO_2_/N_2_ selectivity of 31–42 in CO_2_/N_2_ mixtures separation. This is a successful attempt for the development of hybrid organosilica membranes in CO_2_ capture. In fact, fabrication parameters, such as calcination temperature, calcination atmosphere, and water/organosilica precursor ratios, also play a vital role in the pore structure properties of organosilica membranes [24,25].

In existing studies, BTESA membranes were fabricated via a classic sol-gel strategy. The effect of calcination temperatures on structural properties, including pore sizes and surface chemistry, was extensively studied. FT-IR, SEM, TG, N_2_ sorption, and gas permeation properties were utilized for the investigation of membranes. Specifically, the CO_2_ capture performance was discussed in detail. BTESA membranes calcined at relatively lower temperatures exhibited attractive advantages in CO_2_/N_2_ separation.

## 2. Experimentation

### 2.1. Preparation of Organosilica Sols and Membranes

In this experiment, BTESA was selected as a precursor and ethanol as a solvent. The BTESA precursor was hydrolyzed and polymerized under the combined action of hydrochloric acid and deionized water for the synthesis of BTESA sols. During the synthesis process, a certain amount of BTESA precursor was fully dissolved in ethanol/HCl mixtures, and then a certain amount of deionized water was added dropwise and stirred at 50 °C for 2 h. In the process of synthesizing hybrid silica sol, the molar ratio of components is BTESA: H_2_O: HCl = 1:240:0.01, and the mass fraction of BTESA in the mixed solution is controlled at 5 wt%. In fact, a large number of experiments in the preparation of the BTESA sols have been conducted. BTESA sols with too high concentration, such as 10 wt%, were always unstable during the synthesis process. The partial precipitation of the BTESA monomer could be found. In comparison, BTESA sols with the concentration of 5 wt% (or lower than 5 wt%) were still transparent after the synthesis process. Hence, the concentration of BTESA sols were controlled at 5 wt%.

In the case of membrane fabrication, firstly, α-Al_2_O_3_ particles with SiO_2_-ZrO_2_ sols as a binder were coated on the α-Al_2_O_3_ support (average pore size: 0.2 μm, Nanjing Tech University, Nanjing, China) with calcination at 550 °C for 30 min. This step was repeated 3–5 times for the fully cover of pinholes and macropores. Secondly, SiO_2_-ZrO_2_ sols (0.5 wt%) was coated on the support with calcination at 550 °C in a tube furnace to form an intermediate layer. Finally, the BTESA sol (0.25 wt%) was coated on the intermediate layer and calcined at 100 °C, 300 °C, and 550 °C for 30 min to obtain a separation layer.

### 2.2. Characterization of Organosilica Films and Powders

Fourier transform infrared (FT-IR) spectrometer (FT-IR, Nicolet iS50, Thermo fisher, Waltham, MA, USA) was used to characterize the structural properties of BTESA materials calcined at different temperatures. Organosilica gel powders were prepared by drying at 50 °C and ground using a mortar. The thermal stability of BTESA materials calcined at different temperatures was characterized by a thermogravimetric analyzer (NETZSCH Co., Free State of Bavaria, Germany). N_2_ sorption isotherms of BTESA materials calcined at different temperatures in N_2_ atmosphere were studied by using a gas adsorption analyzer (Micromeritics ASAP 2020, Micromeritics Corporation, Norcross, GA, USA) at −196 °C for the analysis of pore structures. BTESA samples were outgassed at 100 °C for 12 h to remove the adsorbed water prior to the measurement of N_2_ sorption. The morphologies and structures of membranes were examined using a field-emission scanning electron microscopy (SEM, SUPRA55, carl zeiss, Oberkochen, Germany).

### 2.3. Gas Permeation Properties of BTESA Membranes

The gas permeation of BTESA membranes was measured by using the experiment equipment which can be found elsewhere [23]. Highly purified gases (He, H_2_, CO_2_, Ar, N_2_, CH_4_, CF_4_, and SF_6_, Huayang Gas Co., Ltd., Changzhou, China) were utilized to probe gas permeation properties of BTESA membranes at different temperatures. He gas with a flow rate of 50 mL min^−1^ was fed into the membrane module to remove any adsorbed water molecule and gas before a gas permeation test. In the case of binary CO_2_/N_2_ separation, the composition of the gas mixtures was determined by the gas chromatography.

## 3. Results and Discussion

### 3.1. Structural Properties of BTESA Materials

**Figure 1:**
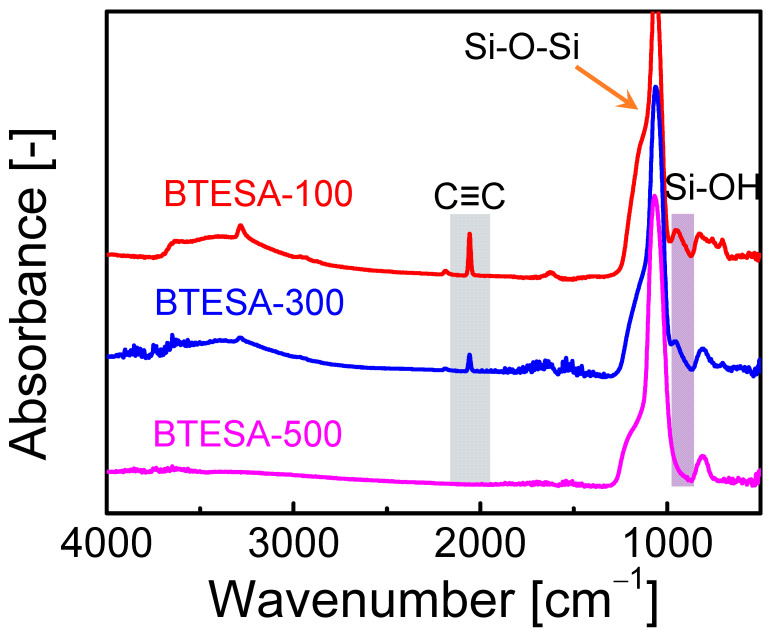
FT-IR spectra of BTESA materials calcined at 100 °C, 300 °C, and 500 °C.

Figure 1 presents the FT-IR spectra of BTESA materials calcined at 100 °C, 300 °C, and 500 °C, respectively. Clearly, the peak intensity of C≡C bonds located at about 2060 cm^−1^ decreased as calcination temperature increased, indicating the pyrolysis of C≡C bonds at high temperatures [26]. The C≡C bond of BTESA-500 disappeared, proving complete decomposition at a calcination temperature of 500 °C. Similarly, the intensity of Si-OH groups was also weakened as calcination temperature increased from 100 °C to 500 °C [27]. This was due to the enhanced condensation reaction between Si-OH groups [24]. The Si-OH groups were formed by the hydrolysis reactions of ethoxy groups within BTESA monomers. Subsequently, the Si-OH groups further reacted with each other and formed the Si-O-Si groups under the high calcination temperatures. Further, the formation of Si-O-Si groups arranged from 1012 to 1063 cm^−1^ for each BTESA material confirmed the existence of amorphous organosilica networks [25]. In a word, the evolution of typical peaks shown in the FT-IR spectra illustrated the effect of calcination temperatures on BTESA networks.

**Figure 2:**
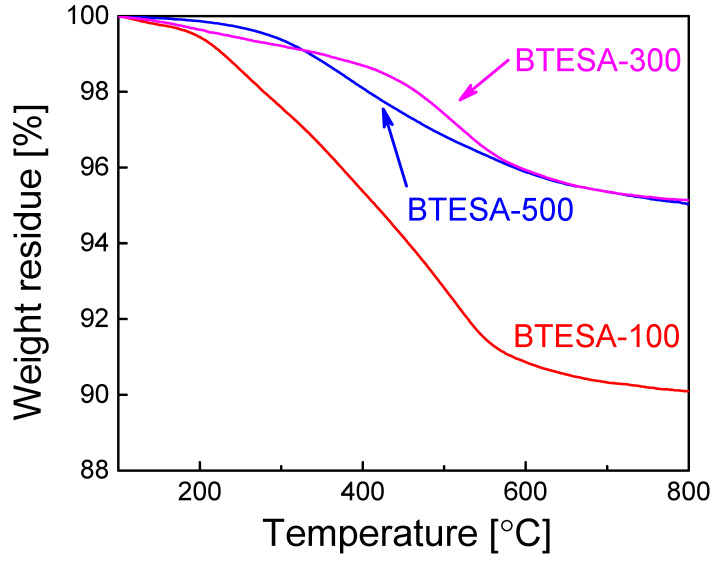
TG analysis of BTESA materials calcined at 100 °C, 300 °C, and 500 °C in the N_2_ atmosphere.

Figure 2 shows a thermal stability test of BTESA powders. It should be noted that BTESA powders were calcined at 100 °C, 300 °C, and 500 °C in the N_2_ atmosphere prior to the measurement of TG, respectively. In the case of the experiment process, BTESA powders were firstly placed in the pan and calcined at 100 °C for at least 1 h to eliminate the effect of adsorbed water molecules. A slow decline of weight residues can be found for each sample before 300 °C, which may be partially attributed to the condensation of the Si-OH groups and the decomposition of C≡C [27]. In addition, it is obvious that weight residues of BTESA-100 and BTESA-300 are more profound than those of BTESA-500. This indicated that the pyrolysis of C≡C bonds already started at the temperature below 300 °C, which is consistent with the characterization results of FT-IR spectra. With the temperature further increased to 800 °C, BTESA-100 featured the lowest weight residue in comparison with BTESA-300 and BTESA-500 samples, which was ascribed to the complete existence of the C≡C bonds under the calcination at 100 °C before the measurement of TG analysis [28].

**Figure 3:**
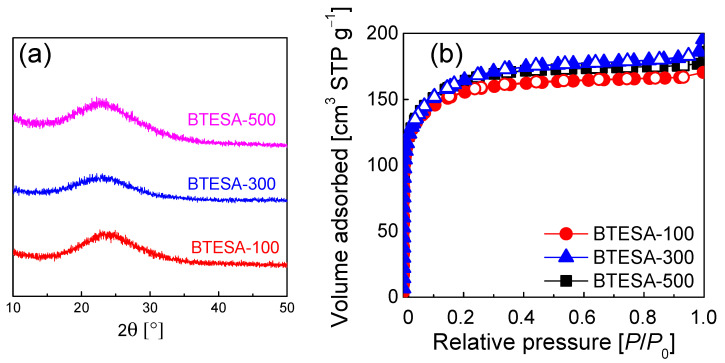
(**a**) The XRD patterns and (**b**) N_2_ sorption isotherms for BTESA-100, BTESA-300 (Data from [23]), and BTESA-500 powders.

Figure 3 presents the XRD patterns and N_2_ sorption isotherms for BTESA-100, BTESA-300, and BTESA-500 powders. Figure 3a shows that all the BTESA samples featured smooth and broad Si-O-Si peaks in the 2θ range of 15°~30°, indicating amorphous network structures of BTESA at different calcination temperatures [29]. The calcination temperatures did not destroy the amorphous structures of BTESA. The N_2_ sorption isotherms presented in Figure 3b show type-I isotherms based on the classification of IUPAC [30], which suggests that the microporous structure of BTESA samples has nothing to do with calcination temperature. The information in Table 1 clearly reflected the evolution of pore structures. As calcination temperature increased from 100 °C to 500 °C, both the pore volume and surface area increased, indicating that the higher calcination temperature is beneficial for the construction of looser structures. The acetylene bridges that had completely degraded at a calcination temperature of 500 °C tended to generate greater porosity in the resultant membrane network, hence, contributing to an increased gas permeance. In other words, the loose structure of BTESA material was constructed under the high calcination temperatures.

**Figure 4:**
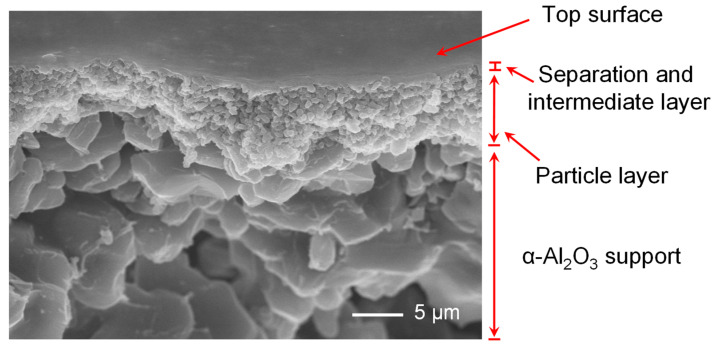
The SEM image of the surface and the cross-section of BTESA-100 membrane.

To further probe the structure of BTESA composite membranes, the BTESA membranes calcined at 100 °C were characterized by FE-SEM, as shown in Figure 4. The composite membranes can be divided into four layers: separation layer, intermediate layer, particle layer, and the alumina support layer from top to bottom [29]. However, it is difficult to clearly distinguish the boundary between the separation layer and intermediate layer due to the thin thickness. The figure shows that the top layer of the BTESA composite membrane is flat and defect-free, which is beneficial for the highly selective CO_2_ separation.

### 3.2. Gas Permeation Properties of BTESA Membranes

**Figure 5:**
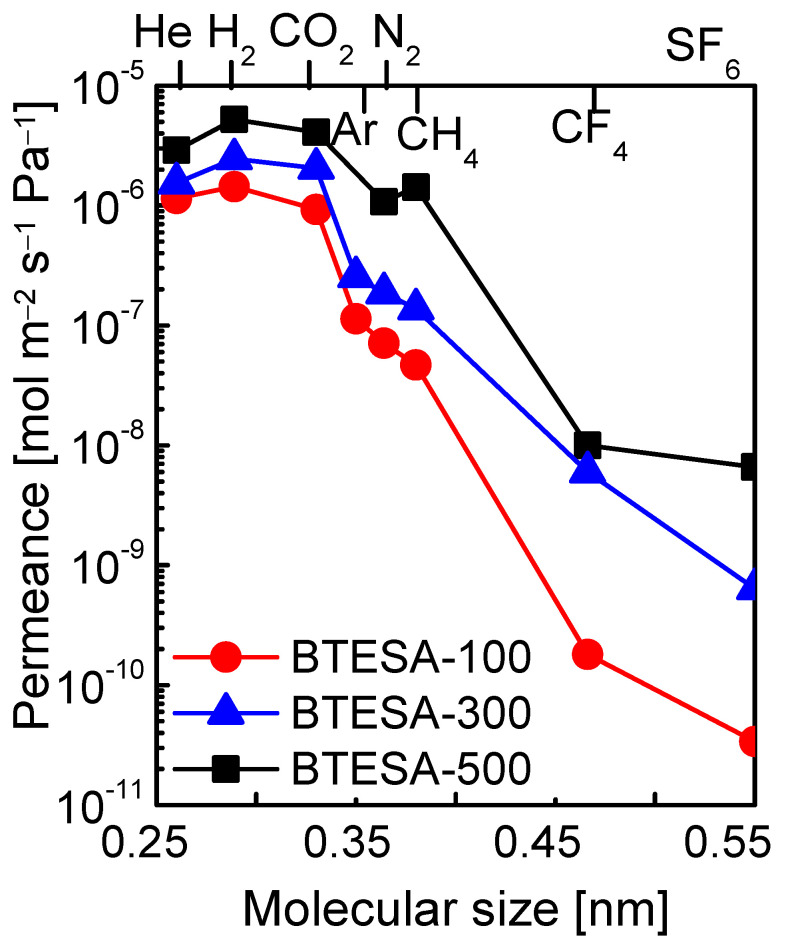
Molecular size dependence of gas permeance at 100 °C for BTESA membranes at different calcination temperatures.

The present study intended to discuss the effect of calcination temperatures on the microstructure and gas permeation properties of BTESA membranes. Hence, the resultant membranes should obtain different structural properties after calcination at different temperatures. BTESA membrane calcined at 100 °C retained all the acetylene bridges and a large number of Si-OH groups. The acetylene bridges partially decomposed, and the number of Si-OH groups further decreased for BTESA-300 membranes. Additionally, BTESA membrane calcined at 500 °C featured complete decomposition of acetylene bridges and condensation of Si-OH groups. The relationship between the structure and the gas permeation properties can be discussed in-depth for BTESA membranes calcined at different temperatures. Figure 5 illustrated the molecular size dependence of gas permeance at the measurement temperature of 100 °C for BTESA-100, BTESA-300, and BTESA-500 membranes. Obviously, the permeance of each gas molecule enhanced as the membrane calcination temperature rose. With an increase in calcination temperature, membrane structures of the traditional silica or BTESE-representative organosilica membranes tended to be more densified due to the enhanced hydrolysis and condensation reactions [24,25]. BTESA obtaining rigid C≡C bonds and a long Si-Si distance exhibited totally different network structures in comparison to silica or BTESE membranes [23,27]. With a rise in the calcination temperature, the partial or complete pyrolysis of rigid C≡C bonds contributed to loose structures. The H_2_/N_2_ selectivity, which can be used to characterize the evolution of membrane pore sizes, decreased for BTESA-100 (20), BTESA-300 (13), and BTESA-500 (4.9) membranes [23,31]. This clearly certified the enlargement of pore sizes with a rise in calcination temperature. For the ideal separation of CO_2_/N_2_, BTESA-100 membranes have the highest CO_2_/N_2_ selectivity around 13, reflecting great potential in CO_2_ capture application. The reproducibility and reliability of gas permeation properties for the BTESA membranes calcined at different temperatures were evidenced by the test of at least 3 membrane samples. The error values for the gas permeation test were controlled within 5%–10%.

**Figure 6:**
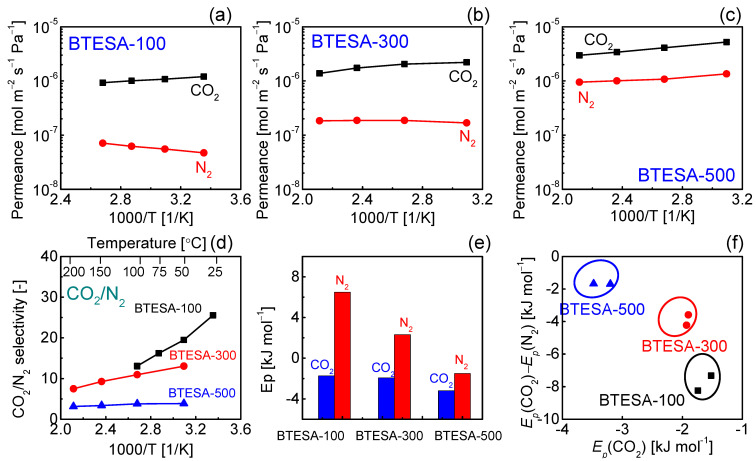
Temperature dependence of gas permeance for (**a**) BTESA-100, (**b**) BTESA-300, and (**c**) BTESA-500 membranes. (**d**) The selectivity of CO_2_/N_2_ at different temperatures. (**e**) The activation energy (*E_p_*) of CO_2_ and N_2_, and (**f**) the relationship between *E_p_*(CO_2_) and *E_p_*(CO_2_)-*E_p_*(N_2_) for BTESA membranes at different calcination temperatures.

The temperature dependent gas permeance of CO_2_ and N_2_ for BTESA membranes calcined at 100 °C, 300 °C, and 500 °C has been presented in Figure 6a–c. BTESA-100 membrane was measured at the temperature from 100 °C to 25 °C. In comparison, the gas permeation properties of BTESA-300 and BTESA-500 membranes were studied within the temperature range of 200 °C to 50 °C. The CO_2_ permeance experienced a rising trend with a decrease in measurement temperature, which indicated that the CO_2_ permeation through the membranes was dominated by the surface diffusion mechanism [21]. Nevertheless, the N_2_ permeation displayed a totally different evolution trend in comparison to the CO_2_ permeation. In the case of BTESA-100 and BTESA-300 membranes, the N_2_ permeance decreased as the temperature decreased. For BTESA-500 membrane, the N_2_ permeance increased as the temperature increased. Different N_2_ permeation behaviors showed that the N_2_ permeation through BTESA-100 and BTESA-300 membranes was governed by the activated diffusion mechanism and that of BTESA-500 membrane was dominated by the surface diffusion mechanism, respectively [21]. The enlarged pore sizes or the generated defects attributed to the decomposed acetylene bridges determined the N2 permeation properties of BTESA-500 membranes. As a result, the CO_2_/N_2_ selectivity presented an enhanced trend as the measurement temperature decreased. Specifically, the evolutional trend of BTESA-100 membrane was more profound than those of BTESA-300 and BTESA-500 membranes. In comparison to BTESA-300 and BTESA-500 membranes, BTESA-100 membrane featured the highest CO_2_/N_2_ selectivity around 26 and a high CO_2_ permeance of 1.2 × 10^−7^ mol m^−2^ s^−1^ Pa^−1^ (3584 GPU) at 25 °C, satisfying the requirement of competitive separation performance for industrial application [22].

Furthermore, as shown in Figure 6e, the activation energies (*E_p_*) of CO_2_ and N_2_ were calculated based on the temperature dependent gas permeance of BTESA-100, BTESA-300, and BTESA-500 membranes [32,33,34]. As the membrane fabrication temperature increased, the activation energies of CO_2_ and N_2_ for each membrane decreased, demonstrating that membrane pores expanded with the increasing calcination temperature. In previous studies, Yu et al. reported that the *E_p_*(CO_2_) and the difference between *E_p_*(CO_2_) and *E_p_*(N_2_) could reflect the potential for CO_2_ permeation and CO_2_/N_2_ separation, respectively [12,35]. As shown in Figure 6f, BTESA-100 membranes featured the largest potential for CO_2_/N_2_ separation, while BTESA-500 membranes displayed the hugest potential for CO_2_ permeation. In fact, the preferred region is located at the left-bottom of Figure 6f, which will be a future target for CO_2_ separation to develop novel organosilica membranes with high potential for CO_2_ permeation and separation.

**Figure 7:**
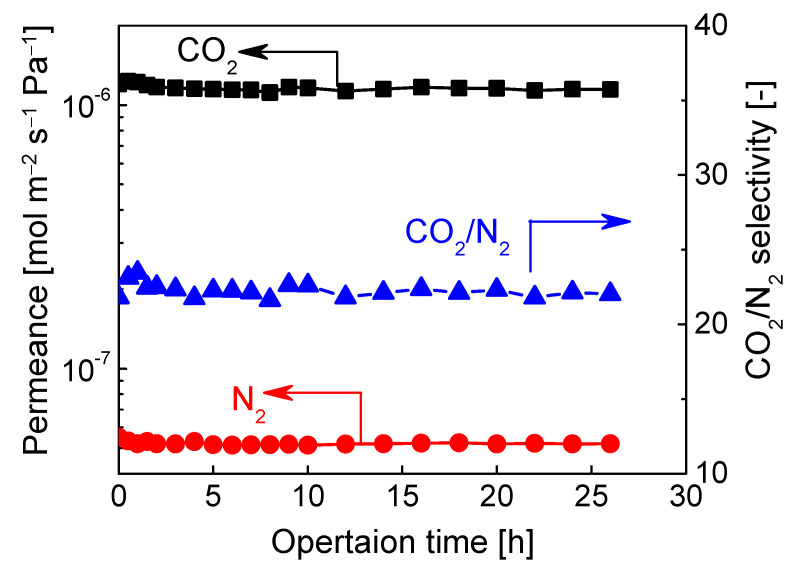
A long-term stability test of CO_2_/N_2_ (15/85) mixtures separation for BTESA-100 membrane at 50 °C.

In terms of CO_2_/N_2_ mixtures separation, BTESA-100 membrane was adopted for analysis measurement. To confirm the stability of its separation performance, a test for the long-term operation stability of binary CO_2_/N_2_ (15/85) separation was conducted at 50 °C, and the results were shown in Figure 7. Clearly, there was no obvious reduction in CO_2_/N_2_ separation performance for both the CO_2_ permeance and CO_2_/N_2_ selectivity in a continuous operation up to 26 h. The BTESA-100 membrane was verified to be reliable in a long-term separation test and have great potential in CO_2_ capture application. Nevertheless, the membrane stability under a humidified condition also needs to be considered due to the existence of moisture in the practical CO_2_/N_2_ separation process.

**Figure 8:**
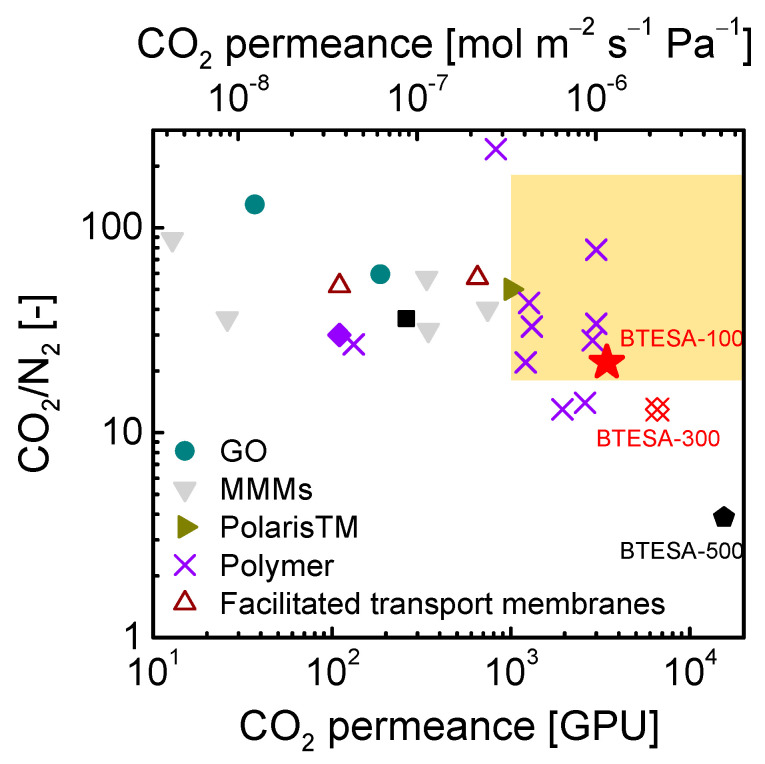
The comparison of CO_2_/N_2_ separation performance between BTESA membranes and other state-of-the-art membranes.

Figure 8 presented the trade-off of CO_2_/N_2_ separation performance between BTESA membranes and other state-of-the-art membranes [14,20,22,36,37,38,39,40,41,42,43,44,45]. The yellow-marked region located in the range of CO_2_ permeance higher than 1000 GPU and CO_2_/N_2_ selectivity higher than 20 represented a target for practical CO_2_/N_2_ separation, as reported by Merkel et al. In contrast to traditional BTESE membranes, which exhibited high CO_2_/N_2_ selectivity around 28 and low CO_2_ permeance of 1 × 10^−7^ mol m^−2^ s^−1^ Pa^−1^ (300 GPU), BTESA membranes at different calcination temperatures displayed unprecedentedly high CO_2_ permeance. As the calcination temperature increased from 100 °C to 300 °C, an exact change in CO_2_/N_2_ separation properties was observed for BTESA membranes. BTESA-100 membrane demonstrated satisfactory CO_2_ permeance of 3434 GPU and CO_2_/N_2_ selectivity of 22 for binary CO_2_/N_2_ (15/85) separation at 50 °C, outperforming most reported membranes in the literature. Moreover, the CO_2_ capture performance of BTESA-100 membrane far exceeded the minimum requirement for industrial application, which may promote the development of novel organosilica membranes for CO_2_ separation.

## 4. Conclusions

In conclusion, the effect of calcination temperature on the structural and gas permeation properties of organosilica membranes have been elucidated in detail. BTESA membranes calcined at low temperatures retained more silanol groups and relatively densified network structures. As the calcination temperature rose from 100 °C to 500 °C, C≡C bridges decomposed and yielded more porosity, which enlarged membrane pore sizes. As a result, the resultant membranes present totally different CO_2_ capture performance. BTESA-300 and BTESA-500 membranes showed high CO_2_ permeance, but low CO_2_/N_2_ selectivity. In contrast, BTESA-100 membrane showed satisfactory CO_2_ permeance of 3434 GPU and CO_2_/N_2_ selectivity of 22 for CO_2_/N_2_ (15/85) mixtures separation at 50 °C, outperforming most reported membranes. This paper studies the effect of calcination temperature on organosilica membranes in detail and may pave the way for the highly efficient CO_2_ capture application.

## Figures and Tables

**Table 1:** The specific pore structure of BTESA powders calcined at different temperatures.

Sample	Surface Area *S* [m^2^ g^−1^]	Pore Volume *V*_p_ [cm^3^ g^−1^]
BTESA-100	513	0.29
BTESA-300	594	0.33
BTESA-500	619	0.34

## Data Availability

Not applicable.
